# Leptin and Acute Lung Disorders

**DOI:** 10.1002/cph4.70025

**Published:** 2025-07-09

**Authors:** Alice Huertas, Jahar Bhattacharya

**Affiliations:** ^1^ Université Paris‐Saclay, School of Medicine DYNAMIC Lab Le Kremlin‐Bicêtre France; ^2^ Vagelos Columbia University College of Physicians and Surgeons New York New York USA

**Keywords:** acute lung injury, acute respiratory distress syndrome, hyperleptinemia, JAK–STAT pathway, obesity paradox

## Abstract

Leptin, an adipokine primarily produced in white adipose tissue, plays a crucial role in metabolism, immunity, and inflammation. Originally identified as a satiety hormone, leptin is also synthesized in various tissues, including the lungs, where it regulates immune responses by binding to the ObR receptor and activating pathways like JAK–STAT3 and PI3K. Functioning as a cytokine‐like hormone, leptin modulates innate and adaptive immunity by promoting T and B cell proliferation, macrophage activation, and chemokine secretion. In lung physiology, leptin contributes to maturation and alveolar development, but its role in acute lung disorders such as acute respiratory distress syndrome (ARDS) remains controversial. The “obesity paradox” suggests that obese patients may be protected against ARDS, potentially due to hyperleptinemia‐driven immune modulation, enhanced neutrophil recruitment, and improved alveolar macrophage function. However, obesity‐induced leptin resistance may impair these protective effects. Conflicting animal studies on leptin's role in acute lung injury (ALI) further complicate its understanding, with some showing protection and others increased susceptibility to lung damage. Further research is needed to clarify leptin's influence on lung inflammation and its interplay with metabolic disorders like obesity, which could inform targeted therapeutic strategies for ARDS and other pulmonary diseases.

## Graphical abstract

Leptin, an adipocyte‐derived cytokine‐like hormone, plays a crucial role in metabolism and immunity but its role in acute lung disorders such as acute respiratory distress syndrome (ARDS) remains controversial. Elucidating leptin's role in lung inflammation, and its interplay with metabolic disorders like obesity, will help designing therapeutical strategies in ARDS.

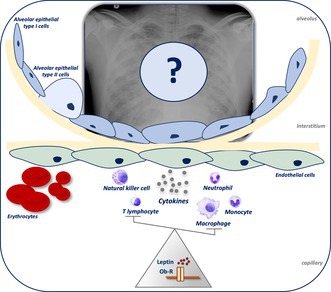

## 1 Leptin Pathway

The adipokine leptin, called from the Greek λεπτός—leptos or thin, is the product of the obese gene (*Ob*; also known as Lep). Mice lacking the gene (*Ob/Ob* mice) were created by positional cloning in 1994 (Zhang et al. 1994). This 16 kDa protein, considered the satiety hormone, regulates feeding behavior through the central nervous system and controls the amount of stored body fat (Hummel et al. 1966). *Ob/Ob* mice show hyperphagia, obesity, insulin resistance and a low resting metabolic rate and administration of leptin to reverses these changes (Pelleymounter et al. 1995; Friedman 2016). Moreover, leptin improves metabolic abnormalities, including insulin resistance and hyperlipidemia, when administrated to lipoatrophic mice which have low levels of leptin due to the lack of subcutaneous adipose tissue (Shimomura et al. 1999). Leptin has also been shown to be effective at improving metabolic dysfunction in patients with lipodystrophy or congenital leptin deficiency (Oral et al. 2002; Farooqi et al. 2002). These hallmark studies established leptin as an adipocyte‐derived hormone essential for the balance between food intake and energy expenditure.

Leptin is mainly produced in white adipose tissue, at least in part controlled by food intake: food consumption results in increased expression of the leptin encoding *Ob* gene, whereas fasting reduces leptin concentrations by decreasing *Ob* expression (Auwerx and Staels 1998). Besides adipose tissue, more recent work has documented that several other cell types can produce leptin in response to a variety of stimuli, such as skeletal muscle, placenta, brain, mammary and gastric fundic epithelium, intestine, bone marrow, and lymphoid tissues (Ahima and Flier 2000). Leptin is also expressed in the lungs, as bronchial epithelial cells, alveolar type II pneumocytes, macrophages, and pulmonary endothelial cells all synthesize leptin (Vernooy et al. 2009; Bruno et al. 2009; Huertas et al. 2012). Leptin levels in the bronchoalveolar lavage correlate with the systemic concentration, suggesting the hypothesis that leptin might also be transported from the blood into the lungs (Holguin et al. 2007).

Interestingly, leptin has structural homology with cytokines of the long‐chain helical family that comprises interleukins (IL)−2, −6, and −11 and hormones such as erythropoietin, thrombopoietin, prolactin, growth hormone, and colony stimulating factors (granulocyte and macrophage) (Hotamisligil 2006) and leptin levels in the serum and adipose tissues are increased in response to pro‐inflammatory stimuli, including tumor necrosis factor (TNF) and lipopolysaccharide (LPS) (Grunfeld et al. 1996). Leptin signals through a type I cytokine receptor called ObR, which is a transmembrane receptor containing a common amino acid motif (WSXWS) in its extracellular domain (Tartaglia 1997; La Cava and Matarese 2004). When leptin binds its receptor, it activates multiple signal transduction pathways, including janus kinase‐signal transducer and activator of transcription‐3 (JAK‐STAT3), phosphatidylinositol 3‐kinase (PI3K), mitogen‐activated protein kinase (MAPK) and 5′ adenosine monophosphate‐activated protein kinase (AMPK), which are implicated in cell differentiation and proliferation. ObR is encoded by the *db* gene and is located predominantly in the hypothalamus and cerebral micro‐vessels that constitute the blood–brain barrier. However, it is also widely distributed in the lungs and expressed in the epithelial cells of the bronchus and alveoli, bronchial submucosa, and bronchial and pulmonary vascular smooth muscle cells (Huertas et al. 2012; Nair et al. 2008; Bruno, Chanez, et al. 2005).

## 2 Leptin and Lung Immunity

Because of its structure and signaling pathway, leptin is now clearly considered as a cytokine‐like hormone. Increasing evidence implicates leptin as a pleotropic hormone, regulating a wide range of systemic physiological functions such as cellular homeostasis and metabolism, glycemic control, neuroendocrine function, angiogenesis, bone formation and reproduction (Morton et al. 2006; Haynes et al. 1997; Bouloumié et al. 1998; Ducy et al. 2000; Chehab et al. 1996). Since ObR is expressed by all cell types involved in innate and adaptive immunity, including neutrophils, monocytes, macrophages, T and B lymphocytes, mast cells, and dendritic cells, it implicates leptin as an important mediator of inflammation (La Cava and Matarese 2004). The widespread distribution of ObR clearly indicates leptin not only as a pro‐inflammatory adipokine, but also to be a master regulator of the immune system and inflammatory responses at the systemic level.

In the lung, leptin‐mediated regulation of both innate and adaptive immunity through the modulation of immune cell survival and proliferation represents an important factor in the maintenance of pulmonary immunity as almost all immune cells express ObR, even if its clinical impact remains to be established (Vernooy et al. 2013). In innate immunity, leptin increases the production of reactive oxidative species and TNF and IL‐6 and promotes cell proliferation and migratory responses (Farooqi et al. 2002; Zarkesh‐Esfahani et al. 2004; Bruno, Conus, et al. 2005). Leptin stimulates the production of CC‐chemokine ligands (namely, CCL3, CCL4 and CCL5) by macrophages by activating the JAK2‐STAT3 pathway, the cytotoxicity of natural killer cells and promotes the activation of granulocytes and dendritic cells (Santos‐Alvarez et al. 1999; Kiguchi et al. 2009; Lam et al. 2006; Zhao et al. 2003).

In adaptive immunity, leptin increases the proliferation and maturation of naïve T and B cells whilst decreasing the inhibitory effects of regulatory T cells on the immune response (Procaccini et al. 2017; Francisco et al. 2018). *Ob/Ob* mice have increased thymocyte apoptosis and diminished thymic cellularity that can be reversed by leptin, showing that leptin is important for thymic homeostasis and maturation (Howard et al. 1999; Lord et al. 1998). Leptin activates T cells or mononuclear cells to secrete pro‐inflammatory cytokines such TNF‐α, IL‐6, IL‐12, IL‐2 and interferon (IFN)‐γ, rather than anti‐inflammatory type 2 T helper cell (Th2) type cytokines, facilitating T cell priming (Francisco et al. 2018), thus polarizing T cells towards a Th1 cell phenotype (Howard et al. 1999; Lord et al. 1998; Martín‐Romero et al. 2000; Maurya et al. 2018). Leptin treatment can lead to a significant increase of CD4^+^ and CD8^+^ T cells, NKT cells, cytokine responsiveness and can promote survival of both T and B lymphocytes, regulates B cell development and activates B cells to secrete cytokines (Oral et al. 2006). Consistent with these findings, leptin deficiency protects against liver damage in models of T cell‐mediated hepatitis (Faggioni et al. 2000). In addition, *Ob/Ob* mice are resistant to the induction of experimental autoimmune encephalomyelitis, owing to the polarization of T cells towards the Th2‐type phenotype rather than the pathogenic Th1‐type phenotype (Matarese et al. 2001).

## 3 Leptin and Lung Disorders

Leptin's role in lung physiology is evident through studies of lung development and maturation. Thus, leptin and its receptor are expressed both in the placenta and in the fetus lungs (Henson et al. 2004), and leptin induces lung maturation and increases the expression of surfactant proteins (Hoggard et al. 1997; Tsuchiya et al. 1999; Kirwin et al. 2006; Chen et al. 2013). Leptin is also critical to postnatal lung remodeling as indicated by the lower alveolar surface area in *ob/ob* mice, which do not increase alveolar size with age or after treatment with leptin 1 month after birth (Huang et al. 2008). Together, these results suggest that leptin regulates fetal and postnatal lung maturity. However, further studies are needed to better understand leptin's role in terms of its therapeutic options for respiratory complications of preterm birth.

**FIGURE 1:**
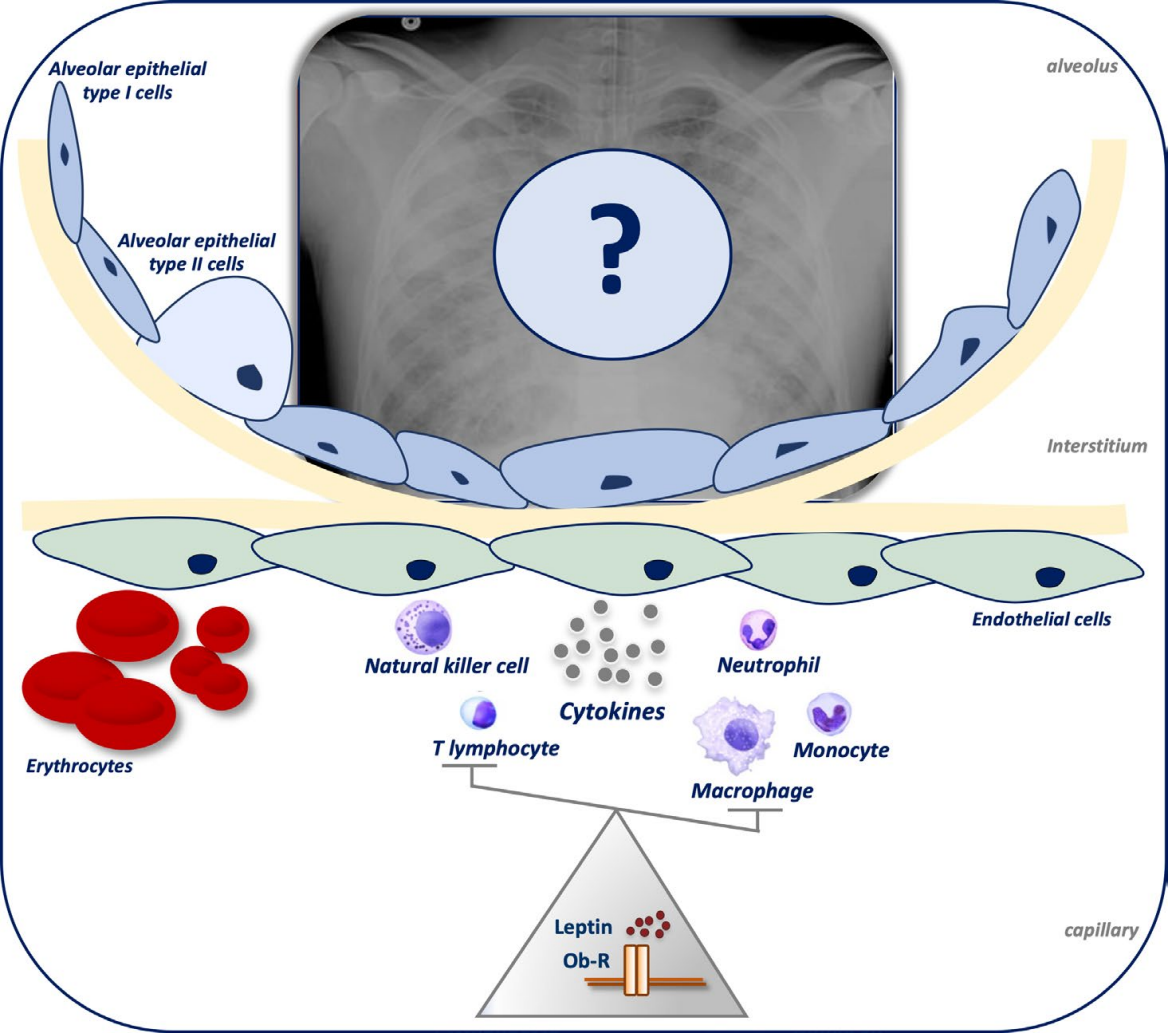
Schematic overview of the role of leptin signaling pathway on cytokines and immune cells involved in acute lung injury pathogenesis. The effect on the different immune cell types of leptin's concentration and its ability to signal through its receptor is still not fully understood. Ob‐R, leptin receptor.

As reviewed by Jutant and others, leptin plays a role in several lung diseases such as asthma, COPD, fibrosis, PH and lung cancer, but its role in acute onset of lung disorders, such as lung infections and acute respiratory distress syndrome (ARDS) remain controversial (Jutant et al. 2021; Malli et al. 2010) (Figure 1).

## 4 Obese Patients Are Protected Against ARDS: Obesity Paradox

One poorly understood phenomenon in obese patients is referred to as the “*obesity paradox*”, in which critically ill obese patients showed better prognosis in ARDS and improved outcomes in acute bacterial pneumonia as compared to non‐obese patients (Rubenfeld et al. 2005; Zhi et al. 2016; Spelta et al. 2018; Ni et al. 2017; Corrales‐Medina et al. 2011; Inoue et al. 2007; LaCroix et al. 1989; Nie et al. 2014; Oliveros and Villamor 2008; Jain et al. 2011; Decruyenaere, Steen, Colpaert, et al. 2020). Other outcome data on the association between obesity and the risk and severity of pneumonia from bacterial infections show that, although obesity is linked to increased risk of hospitalisation (Kornum et al. 2010), it also acts as a protective factor during infections (Corrales‐Medina et al. 2011; Singanayagam et al. 2013). Multiple hypotheses have emerged over the years without shedding light on the underlying mechanisms. One consideration is that following infection, congenitally leptin‐deficient patients and leptin‐deficient mice show increased mortality that is reversed by leptin therapy (Farooqi et al. 2002). Therefore, since adipose tissue is the main source of leptin (Trayhurn 2013; Grosfeld et al. 2002; Palhinha et al. 2019), it is possible that adipose tissue‐derived hyperleptinemia underlies the protection in obese patients.

It is also known that adipocytes are able to activate monocytes, macrophages, and dendritic cells and to secrete not only pro‐inflammatory factors but also numerous adipocytokines, such as adiponectin, that play a vital role in the secretion of anti‐inflammatory cytokines such as IL‐10 and IL‐1 receptor antagonist, and such as leptin, which appears central in the host defense (Weisberg et al. 2006; Ouchi et al. 2011). Indeed, leptin plays an important role in the recruitment of neutrophils to sites of infection in the lung by exerting a direct chemotactic effect and may further increase neutrophil numbers in the airspace by inhibiting neutrophil apoptosis (Ubags et al. 2014) and by increasing reactive oxygen species production, such as hydrogen peroxide (Fantuzzi 2005). Interestingly, it has been recently demonstrated that leptin signaling in the airways can ameliorate acute lung injury through the role of its receptor ObR. Alveolar macrophages also specifically express the leptin receptor ObR, and the inherent ObR signal of alveolar macrophages seems to play a protective role in pulmonary inflammation by preventing excessive lipid droplet formation and alleviating metabolic stress in the fat‐rich alveolar microenvironment. In the work by Guo and coworkers, the ObR signal has been shown to maintain adenosine monophosphate‐activated protein kinase activation in a calcium influx‐dependent manner and to restore cellular metabolism, defining ObR‐expressing alveolar macrophages as a metabolic checkpoint of pulmonary inflammation (Guo et al. 2022).

Another consideration regarding the obesity paradox is the recognition that adipose tissue is not only an energy storehouse, but it also plays a role in immune regulatory function. Thus, obesity may cause a macrophage‐induced chronic low‐grade inflammation in adipose tissue marked by the secretion of inflammatory cytokines, such as TNF‐α, IL‐6, and IL‐1, from adipocytes and resident adipose tissue macrophages (Ervin 2009; Weisberg et al. 2003). This chronic low‐grade inflammatory status has been thought to create a protective environment, limiting the detrimental effects of a more aggressive second hit, such as sepsis or ventilator‐induced lung injury (Bustamante and Repine 2013).

Leptin is one of the pro‐inflammatory cytokines released by the adipose tissue. Although obesity is associated with hyperleptinemia (Trayhurn 2013; Grosfeld et al. 2002; Palhinha et al. 2019), obese patients frequently develop central and peripheral leptin resistance (Frederich et al. 1995) that limits leptin's ability to be effective in target cells due to reduced ObR expression or disturbed ObR signaling. This ineffectiveness is evident in the finding that, despite high blood levels of leptin obese individuals do not display the expected anorexic responses (Friedman and Halaas 1998). A major exception to the predictions of the obesity paradox is the increased obesity‐associated mortality after severe SARS‐CoV2 infection (Peters et al. 2018; Bansal et al. 2020; Korakas et al. 2020; Chu et al. 2020). Clearly therefore, much needs to be understood regarding the roles of obesity and leptin signaling in the lungs (Maurya et al. 2021), especially whether the obesity paradox results from a direct deleterious effect of increased levels of leptin or from leptin resistance.

The role of leptin in acute lung injury (ALI) is controversial in animal models as well: mice lacking leptin or its receptor were described as having many defects in cell‐mediated and humoral immunity but some studies show that mice deleted in ObR develop less lung injury (Kordonowy et al. 2012; Mancuso et al. 2002) and display better survival in a model of ALI/ARDS induced by hyperoxia exposure (Bellmeyer et al. 2007), whereas others demonstrate a protective role of leptin with high susceptibility to inhaled endotoxin‐induced lethality in leptin‐deficient mice, which can be reversed by the administration of leptin (Faggioni et al. 1999; Dong et al. 2013; Hsu et al. 2007).

All the studies described above focus on leptin deficiency, receptor and signaling defects or conditions of starvation and not obesity per se, so further studies are needed to shed light on the obesity‐related lung in particular in lung infections.

## 5 Conclusion

Leptin appears to be central in controlling pulmonary immune homeostasis, in particular in acute lung inflammation, but epithelial‐endothelial crosstalk due to leptin needs to be better understood. Further studies should investigate the connection between leptin signaling and pulmonary cells, in particular in the context of metabolic disorders like obesity. The etiology of ARDS seems to be of importance for mechanistic aspects of host defense. The underlying mechanisms of acute lung inflammation differ whether the injury is mediated by oxidative burst leading to cell membrane disruption or by toll‐like receptor (TLR) 4‐responses and altered alveolar defense mechanisms.

Taken altogether, the apparent discrepancies in the literature about the role of leptin may rely on the cell‐specificity of the overall signaling pathway and the organ concerned. Clarifying controversies about the role of leptin in acute lung inflammation will help design therapeutical strategies in ARDS.

## Conflicts of Interest

The authors declare no conflicts of interest.

## Data Availability

Data sharing is not applicable to this article as no new data were created or analyzed in this study.
